# Development of a Recombinase-Mediated Cassette Exchange System for Gene Knockout and Expression of Non-Native Gene Sequences in *Rickettsia*

**DOI:** 10.3390/vaccines13020109

**Published:** 2025-01-22

**Authors:** Benjamin Cull, Nicole Y. Burkhardt, Benedict S. Khoo, Jonathan D. Oliver, Xin-Ru Wang, Lisa D. Price, Kamil Khanipov, Rong Fang, Ulrike G. Munderloh

**Affiliations:** 1 Department of Entomology, College of Food, Agricultural and Natural Resource Sciences, University of Minnesota, St. Paul, MN 55108, USA; burkh032@umn.edu (N.Y.B.); wangxin@upstate.edu (X.-R.W.); pric0129@umn.edu (L.D.P.); munde001@umn.edu (U.G.M.); 2 Division of Environmental Health Sciences, School of Public Health, University of Minnesota, Minneapolis, MN 55455, USA; khoo0011@umn.edu (B.S.K.); joliver@umn.edu (J.D.O.); 3 Department of Pharmacology and Toxicology, University of Texas Medical Branch, Galveston, TX 77555, USA; kakhanip@utmb.edu; 4 Department of Pathology, University of Texas Medical Branch, Galveston, TX 77555, USA; rofang@utmb.edu; 5 Center for Biodefense and Emerging Infectious Diseases, University of Texas Medical Branch, Galveston, TX 77555, USA

**Keywords:** *Rickettsia*, *Anaplasma*, live-attenuated vaccine, transposon mutagenesis, genetic tools

## Abstract

Background/Objectives: Incidence of vector-borne diseases, including rickettsioses and anaplasmosis, has been increasing in many parts of the world. The obligate intracellular nature of rickettsial pathogens has hindered the development of robust genetic tools for the study of gene function and the identification of therapeutic targets. Transposon mutagenesis has contributed to recent progress in the identification of virulence factors in this important group of pathogens. Methods: Combining the efficiency of the himar1 transposon method with a recombinase-mediated system, we aimed to develop a genetic tool enabling the exchange of the transposon with a cassette encoding non-native sequences. Results: This approach was used in *Rickettsia parkeri* to insert a himar1 transposon encoding fluorescent protein and antibiotic resistance genes for visualization and selection, flanked by mismatched *loxP* sites to enable subsequent recombinase-mediated cassette exchange (RMCE). RMCE mediated by a plasmid-encoded Cre recombinase was then employed to replace the transposon with a different cassette containing alternate fluorescent and selection markers and epitopes of *Anaplasma phagocytophilum* antigens. The resulting genetically modified *R. parkeri* was trialed as a live-attenuated vaccine against spotted fever rickettsiosis and anaplasmosis in mice. Conclusions: The use of this system provides a well-established and relatively efficient way of inserting non-native sequences into the rickettsial genome, with applications for the study of gene function and vaccine development.

## 1. Introduction

The incidence of diseases caused by arthropod-borne pathogens has been increasing world-wide, facilitated by the expanding range of their vectors, which is in turn aided by a changing climate. Among these, rickettsioses are particularly challenging to control due to difficulties in diagnosis and a lack of vaccines. Rickettsioses occur worldwide and are caused by a diverse array of *Rickettsia* species (Rickettsiales: Rickettsiaceae), with a similarly diverse spectrum of pathogenicity ranging from mild, self-limiting illness to fatal infections [1]. Since rickettsiae are primarily transmitted through the bites of ticks, fleas, mites, and lice, the main prevention methods focus on reducing contact with arthropod vectors. The diagnosis of rickettsial diseases is complicated by non-specific symptoms and the reliance on serological methods, which, due to the cross-reactivity of antibodies to multiple *Rickettsia* species, means the causative agent is rarely identified [2]. These complications often result in delayed treatment and whilst rickettsioses can be effectively treated with antibiotics, the options are limited to a few antibiotic classes, meaning the development of antibiotic resistance is a future threat to successful treatment, a risk heightened by the current lack of any effective vaccines [3]. Despite the severity and potentially high mortality rates of human rickettsioses, there has been little progress towards alleviating these shortcomings due, in part, to the obligate intracellular nature of the causative bacteria, which is further complicated by technically demanding requirements for their safe handling in the laboratory. *Rickettsia rickettsii*, the tick-borne agent of Rocky Mountain spotted fever, and flea-borne *Rickettsia typhi*, as well as louse-borne *Rickettsia prowazekii*, must be handled under BSL3 containment, restricting research with them to appropriately equipped facilities, which are expensive and not readily available. Due to a combination of these factors, progress in rickettsial genetics has been slow and inconsistent [4,5]. This is a major impediment to the identification of gene function in rickettsial pathogens where more than one third of the genome consists of hypothetical genes. Robust genetic systems for Rickettsiales would facilitate the discovery of unique metabolic pathways that could be targeted for the development of novel therapeutics, and aid in producing effective vaccines [6].

Several strategies have been attempted for the development of rickettsial vaccines, including inactivated whole organisms, subunit vaccines, and live attenuated vaccines, although each has its own disadvantages and none have been successfully developed beyond the experimental stage [7,8]. Additional experimental rickettsial vaccine approaches include recombinant proteins or peptides, DNA or mRNA-based vaccines, antigen-coupled nanoparticles, bacterial or adenovirus vector-based vaccines, and immunization with antigen-presenting cells [7]. Live-attenuated vaccines are considered one of the most promising approaches due to their rapid onset of immunity and durable protection. Use of transposon-based systems for the random mutagenesis of rickettsial pathogens can generate libraries of mutants that can form a basis for the selection of attenuated mutants for vaccination studies [6,9]. This methodology has been applied to spotted fever group (SFG) rickettsiae, in particular *Rickettsia parkeri* [6,10], which cause mild disease in humans but induce cross-protective immunity to severe pathogens such as the related agent of Rocky Mountain spotted fever, *R. rickettsii* [11,12,13].

We showed that *R. parkeri* transposon mutants with an insertion in a phage integrase gene when injected into C3H/HeN mice induced solid protection against challenge with the wild-type strain and, moreover, protected mice against the Mediterranean spotted fever agent, *Rickettsia conorii* [6]. In addition, we had previously been able to induce the expression of a foreign gene in rickettsiae transformed with shuttle plasmids [14]. Based on these successes, we considered that it should be possible to design a genetic tool that would combine the efficiency of the himar1 transposon system with the ability of a recombinase-mediated system for the facile exchange of the transposon with a cassette encoding non-native sequences. To test this idea, we designed a himar1 transposon encoding a fluorescent protein gene and an antibiotic resistance gene to facilitate visualization and selection, flanked by mismatched *loxP* sites to enable subsequent recombinase-mediated cassette exchange (RMCE). In this system, a Cre recombinase catalyzes recombination between specific DNA sequences (*loxP* sites) in a donor plasmid and the intragenically inserted transposon, resulting in the excision and replacement of the transposon sequence with the sequence from the plasmid [15]. Mismatched *loxP* sites ensure the desired orientation of the sequence of interest. This approach would allow cassettes encoding desired sequences to be incorporated into the Rickettsiales genome using RMCE. In order to explore the potential of this approach, we designed RMCE cassettes encoding an alternate fluorescent protein gene and selectable marker as well as B-cell epitopes of *Anaplasma phagocytophilum* antigens. These were flanked with the same mismatched *loxP* sequences as the modified himar1 transposon. We reasoned that the marker genes would allow the visualization and enrichment of rickettsiae that had successfully undergone RMCE, and antigenic epitopes from a different Rickettsiales bacterium would allow us to test if these could be correctly expressed, i.e., in such a way that they would be recognized by polyclonal immune sera. By incorporating epitopes of *A. phagocytophilum*, another tick-transmitted member of the Rickettsiales (Family Anaplasmataceae) responsible for human and animal disease worldwide [16] and sharing 80–86% homology with SFG *Rickettsia* species [17], we hoped to provide proof of concept that producing a live-attenuated *Rickettsia* vaccine candidate providing protection from multiple tick-borne diseases is feasible.

Here, we show that rickettsiae can be manipulated to undergo transposon mutagenesis with subsequent RMCE to replace the transposon with a completely different cassette. An advantage of the RMCE reaction is that the cassette accepts and introduces a much larger payload than the himar1 transposon. Use of this system provides a well-established and relatively efficient way of inserting non-native sequences into the rickettsial genome.

## 2. Materials and Methods

### 2.1. Bacterial Strains and Cell Types Used

All experiments were performed using *R. parkeri* Tate’s Hell and *A. phagocytophilum* HGE1. *Rickettsia parkeri* were cultured in ISE6 or Vero cells using standard culture conditions [18,19]. *Anaplasma phagocytophilum* were grown in HL60 cells using established methods [20].

### 2.2. Production of Rickettsia parkeri Mutant Library and Replacement of Transposon Cassette

**Figure 1:**
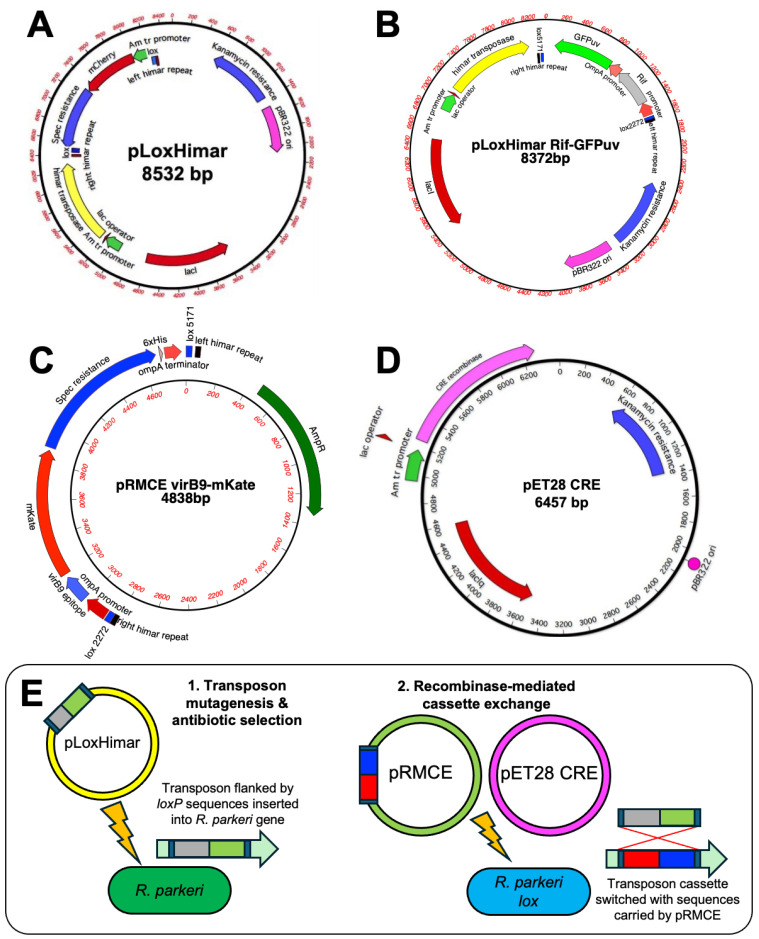
Transposon mutagenesis and recombinase-mediated cassette exchange in *Rickettsia parkeri.* (**A**,**B**) pLoxHimar plasmids designed for transposon mutagenesis of rickettsiae with (**A**) mCherry and spectinomycin/streptomycin resistance or (**B**) GFPuv and rifampicin resistance. (**C**,**D**) Plasmids for (**C**) recombinase-mediated cassette exchange (RMCE) and (**D**) expression of the Cre recombinase. (**E**) Schematic showing the process of transposon mutagenesis followed by insertion replacement by RMCE.

The pLoxHimar plasmid was designed for transposon mutagenesis of rickettsiae, based on our previous successful use of this method in multiple Rickettsiales bacteria [6,9,21,22]. The well-characterized *Anaplasma marginale tr* promoter [23] was used to drive the co-expression of a fluorescent reporter (mCherry) and *aadA* encoding spectinomycin and streptomycin resistance (Figure 1A). Constructs encode both transposase and transposon configured to prevent mobilization of the transposase. The plasmid includes mismatched *loxP-lox5171* and *loxP-lox2272* sites (designated “lox”) flanking the mCherry and *aadA* genes to facilitate excision of the transposon [6]. An alternative construct with GFPuv and rifampicin resistance (*rif*) genes [24] in place of mCherry and *aadA* was also made (Figure 1B) and partial *R. parkeri* libraries were constructed with both of these lox constructs as follows: *R. parkeri* were purified from one 25 cm^2^ flask of ISE6 tick cells using mechanical lysis and differential centrifugation [25]. They were incubated on ice in 50 µL of 300 mM sucrose for 15 min with 1 µg pLoxHimar plasmid DNA, electroporated at 2 kV, 25 µF and 400 Ohm using a Gene Pulser II (BioRAD, Hercules, CA, USA), and recovered in 100 µL fetal bovine serum (FBS). The bacteria were mixed with ~5 × 10^6^ ISE6 tick cells and centrifuged at 5000× *g* for 5 min at room temperature. Centrifuged preparations were left at room temperature for 30 min, and then diluted into 96-well plates pre-seeded with a 50% confluent layer of ISE6 cells at 100 µL/well using L15B300 supplemented with 10% FBS. Antibiotics for selection were added 24 h later in 100 µL of medium to achieve a concentration of 100 µg/mL, and cultures were monitored by fluorescence microscopy to detect marker-expressing rickettsiae. Contents of positive wells were amplified for insertional analysis, as described previously [6], and storage in liquid nitrogen. ﻿Initially, insertion sites were determined by sequencing rickettsiae from positive wells with primers “Ch Up & Out” and “Spec down & out” [26] for pLoxHimar-transformed *R. parkeri* or “Rif up & out” [GACCTTCAAGACCAGATAGTGAC] and “GFPuv down & out” [AACGAAAAGCGTGACCAC] for pLoxHimar Rif-GFPuv-transformed *R. parkeri*, which amplify outwards from either end of the transposon into the interrupted gene. For later mutants, insertion sites were determined by whole genome sequencing. Genomic DNA from mutants were pooled in groups of six and whole genome sequencing was performed at the University of Minnesota Genomics Center. Ten Nextera XT DNA libraries were created and then sequenced on a MiSeq 2×250-bp v2 run. Sequencing data were analyzed using MacVector Assembly program. After characterization of desirable mutants, the *GFPuv* and *rif* genes were replaced with a cassette containing *A. phagocytophilum* epitopes, mKate and *aadA*, by RMCE. To do this, *R. parkeri* mutants were electroporated simultaneously with a Cre recombinase-encoding plasmid and pRMCE carrying the poly-epitope sequences, mKate and *aadA*, flanked by the same *lox* sites (Figure 1C–E). Loss of *rif* was verified by PCR and exposure of rickettsiae to spectinomycin, alongside wild-type *R. parkeri* as a control. Successful RMCE will result in the replacement of GFPuv with mKate (Figure 1E).

### 2.3. Identification of Attenuated R. parkeri Mutants

Several *R. parkeri* transposon mutants were assessed for their ability to cause infection in mice. To determine optimal doses for challenge, a range of doses of WT *R. parkeri* from 10^2^ to 10^7^ were injected intraperitoneally (i.p.) into C3H/HeJ mice (3 age-matched females per group; Jackson Laboratory, Bar Harbor, ME). This mouse strain has been shown to be a good model for SFG rickettsial infections [27,28] and anaplasmosis [29,30]. Mice were weighed daily, and percentage body weight loss was calculated. Based on results of dosage testing with WT *R. parkeri*, each purified transposon mutant was inoculated into two mice at a non-lethal dose of 10^3^ rickettsiae and weights and tissue loads compared with those of mice inoculated with the same dose of WT *R. parkeri*. Mice were euthanized on days 7 and 14 post infection (p.i.), and samples of organs (heart, liver, lung, spleen) were frozen at −20 °C for DNA extraction and qPCR to determine rickettsial loads. DNA was extracted from organs using QIAGEN DNeasy blood and tissue kit (QIAGEN, Hilden, Germany) following manufacturer’s instructions. DNA concentration was adjusted so that each well contained an equal amount of DNA, and qPCR was carried out with primers against the citrate synthase (*gltA*) gene [31], a single-copy gene in SFG rickettsiae [32], using Agilent Brilliant II SYBR Green qPCR master mix (Agilent) on an Mx3000P qPCR System (Stratagene, La Jolla, CA, USA) with cycling conditions as follows: 95 °C 10 min; 40 cycles of 95 °C 30 s, 58 °C 60 s, 72 °C 30 s; and a final dissociation curve of 95 °C 60 s, 58 °C 30 s, 95 °C 30 s. Tissue DNA samples were quantified against a standard curve of 10-fold serially diluted plasmid containing *R. massiliae* citrate synthase. Water was used as a negative control, and all samples were run in triplicate.

### 2.4. Selection of Antigens and Production of Multi-Epitope Arrays

Epitopes were selected from *A. phagocytophilum* genes encoding known or predicted surface-exposed antigens, including epitopes of the type IV secretory system (T4SS) component VirB9-1 (hereafter virB9), and the surface proteins Asp55 and Asp62 [33,34,35]. VirB9 is conserved among *A. phagocytophilum* strains infecting humans, dogs, and rodents in the US, and differs minimally in strains infecting horses or deer [36]. VirB9 is predicted to have a signal peptide and two transmembrane helices; it localizes to the periplasm and surface and is considered a vaccine target [37]. Asp55 and Asp62 have multiple transmembrane domains and surface-exposed loops [35]. It has been shown that conserved subdominant antigens are suitable vaccine candidates and that components of the T4SS are immunogenic, likely due to their location in the outer membrane [33,37]. Additionally, the rickettsial YchF gene encoding a ribosome-binding ATPase was included in the first iteration of epitope array designs because of its expected role in enhancing transcription.

B- and T-cell epitopes from these putative antigens were predicted using Bepipred-2.0 Linear Epitope Prediction [38]; Parker Hydrophilicity Prediction [39]; Kolaskar and Tongaonkar antigenicity scale [40]; Karplus and Schulz flexibility scale [41]; Emini surface accessibility scale [42]; and Chou and Fasman beta turn prediction [43] (Table 1). B-cell epitopes were selected for the following experiments, because antibodies are the most important mediators of immunity during secondary challenge.

Epitope-coding regions were synthesized as chimeric fusion proteins by GenScript (Piscataway, NJ, USA) with multiple configurations, i.e., with different epitopes included or arranged in a different order, then cloned into plasmid vectors for transformation of *Escherichia coli* and *R. parkeri*. Epitopes are separated by linkers: S/G P S/G P S/G.

### 2.5. Expression of Epitope Arrays in E. coli

Epitope arrays were ligated into pET-29a(+) plasmids (Novagen, EMD Millipore, Burlington, MA, USA), then transformed into *E. coli* BL21(DE3) competent cells (New England Biolabs, Ipswich, MA, USA; C2527). Transformed cells were cultured at 37 °C in 2xYT broth and protein expression was induced by addition of 1 mM IPTG for 3 h. Cell pellets were washed in cold PBS then lysed in RIPA buffer (ThermoFisher, Waltham, MA, USA). The lysate was centrifuged, and the supernatant was recovered and boiled for 4 min in 2× Laemmli buffer (BioRAD). Equal amounts of protein (standardized to an equivalent of OD_600_ of bacterial cultures) were run on BioRAD Any kD Mini-PROTEAN TGX precast gels at 95 V for 1 h alongside PageRuler pre-stained protein ladder (ThermoFisher) to determine molecular weight. Separated proteins were transferred to a PVDF membrane (EMD Millipore) by wet transfer at 80 V for 1 h. The membrane was blocked for 1 h at RT with PBST + 5% BSA and probed with anti-6HisTag monoclonal antibody conjugated to horseradish peroxidase (ThermoFisher, MA1-21315-HRP) diluted 1:1000 in PBST + 1% BSA for 1 h at RT. Protein detection was carried out with metal-enhanced DAB substrate kit (ThermoFisher, 34065). A second gel was stained with 0.25% Coomassie blue to visualize total protein.

### 2.6. Epitope Antibody Production in Mice

Protein extracts from transformed *E. coli* were run on 15% wide-well SDS-polyacrylamide gels for 2 h at 70–80 V to separate proteins. A strip of 5–7 mm corresponding to molecular weight of the epitope of interest was excised from the gel with a scalpel. Protein was purified from gel slices using a modification of a Thermo Scientific protocol [44]. Gel slices were placed into a 2 mL tube with 500 µL of elution buffer (50 mM TrisHCl, 150 mM NaCl, 0.1 mM EDTA, pH 7.5). The gel and buffer were then passed 5–6 times between two 5 mL syringes to mash the gel. The gel and buffer were incubated overnight at 30 °C shaking at 225 rpm, then centrifuged at 10,000× *g* for 10 min. Supernatant containing eluted protein was pipetted into a new tube. Protein concentration was determined using the BCA protein assay (ThermoFisher).

Purified epitope peptide was used to immunize mice. Twenty-four C3H/HeJ mice were divided into six treatment groups, each containing four animals: one group for each of the five epitope arrays plus one control group. Injections were prepared using 400 µL TiterMax Gold adjuvant (Sigma Aldrich, Burlington, MA, USA) plus protein in 400 µL elution buffer. Control injections contained adjuvant plus elution buffer only. Emulsions were prepared by adding 200 µL aqueous antigen to 400 µL adjuvant in a 2 mL glass syringe with a 22 G needle and mixing by squeezing in and out of a 1.5 mL tube. When a meringue-like emulsion was formed, the remaining 200 µL antigen was added to the syringe and mixed until completely emulsified. The resulting mixture was divided between four 1 mL syringes to give 4 × 200 µL doses of approximately 50 µg each. Each mouse was injected subcutaneously at four different sites with a 23 G needle. Booster injections of 50 µg, prepared as above, were given four weeks later (day 28 after first immunization). No adverse effects were noted in any mice injected with the recombinant peptide arrays. Two weeks after the booster (day 42), two mice from each group were euthanized with CO_2_ and blood was collected by cardiac puncture using a 1 mL syringe with 25 G needle. On day 47 after first immunization, the remaining two mice in each group were challenged with *A. phagocytophilum* (groups 1–5; 200 µL *Ap*HGE1-infected HL-60 cells in RPMI 1640 + 10% FBS, taken from a culture of 1.6 × 10^5^ cells/mL with 70–80% *Ap* infection rate) or *R. parkeri* (group 6; 200 µL cell-free *R. parkeri* in PBS. *Rickettsia parkeri* from purified bacteria preparations were diluted 1:10,000 in PBS, then 20 µL of this were added to 180 µL PBS to make the 200 µL injected). Mice were injected i.p. using a 1 mL syringe fitted with a 25 G needle. Mouse weights were recorded each day following infection. On day 8 post infection, mice were euthanized by CO_2_ asphyxiation and their blood, liver, lungs, and spleen were harvested for quantification of bacterial loads by qPCR targeting rickettsial *gltA* (as described above) or *Anaplasma 16S RNA* using PER5/6 primers [20].

### 2.7. Expression of Epitope Arrays in R. parkeri

Epitope arrays under control of the *Amtr* promoter were inserted into pRAM18dGSK plasmids [14] and transformed into *R. parkeri* Tate’s Hell. Transformed bacteria were cultured in ISE6 cells and selected with spectinomycin/streptomycin, as described previously [19]. Subsequently, further modifications were made to the array cassette, including replacing the *Amtr* promoter with the *ompA* or *ompB* promoter for improved expression, deletion of YchF sequences, and adding an mKate fluorescent marker for enhanced detection. Various configurations of these improved arrays were inserted into pRAM18dGSK plasmids for transformation of *R. parkeri.* The transformed bacteria were cultured in ISE6 and Vero cells and selected with spectinomycin/streptomycin at 50 μg/mL. Successful transformation and expression of epitope arrays were then assessed using fluorescent microscopy, PCR, RT-PCR, and Western blotting as described below.

Low subpassage *R. parkeri* G8::lox mutant (with a loxHimar insertion in the RPATATE_1142 gene encoding an N-acetylmuramoyl-L-alanine amidase family protein) transformed with pRAM18dGSK plasmids containing various epitope combinations fused to an mKate fluorescent protein, and *R. parkeri* G8::lox mutants with the epitope-mKate cassette swapped into the himar insertion site via RMCE were grown in ISE6 cells. Successful transformations were confirmed by antibiotic selection, fluorescent microscopy, PCR, RT-PCR, and Western blotting.

Genomic DNA was made from all *R. parkeri* transformants with the PureGene Core Kit A (QIAGEN) as per the manufacturer’s protocol for gram negative bacteria. *Rickettsia parkeri* transformed with pRAM18dSGK[epitopes] were tested for the presence of the epitope cassette with primers M13F/MCS193R (Table S1) and GoTaq DNA Polymerase (Promega) in standard PCR reactions with the following cycling parameters: 1 cycle at 95 °C for 2 min; 40 cycles at 95 °C for 30 s, 50 °C for 30 s, 1 min at 72 °C; and a final 7 min period at 72 °C.

The *R. parkeri* G8::lox mutant that had undergone RMCE with the Cre plasmid (Figure 1D) and pRMCE constructs containing various epitopes fused to mKate and the Spectinomycin resistance gene flanked by the inverted lox repeat sites (Figure 1C) were tested for purity with the primer sets RPATATE_1142 F2/ ID2, RPATATE_1142 F2/ rif Up & Out, and RPATATE_1142 F2/ mKATE Up & Out2 using Q5High Fidelity DNA Polymerase (New England Biolabs) as per the manufacturer’s protocol with the following cycling parameters: 1 cycle 98 °C for 30 s, 40 cycles of 98 °C for 10 s, X °C for 30 s, and 72 °C for 30 s, with a final extension step of 2 min at 72 °C, where X is 66 °C, 65 °C, or 67 °C for each primer set, respectively.

*R. parkeri* transformed with pRAM18dSGK[epitopes] were extracted from host cells as described above and put into RNAProtect (QIAGEN) as per manufacturer’s protocol, except pellets were recovered from RNAProtect at 5000× *g* for 15 min to ensure collection of rickettsiae. Recovered pellets were resuspended in 1 mL TRIzol (Thermo Fisher Scientific, Waltham, MA, USA) and vortexed, and RNA was prepared using the Direct-zol RNA MiniPrep kit (Zymo Research, Irvine, CA, USA) as per manufacturer’s protocol. RNA was treated with gDNA Eraser (TaKaRa, San Jose, CA, USA) to remove genomic DNA, and 100 ng of resulting RNA was added to qRT-PCR Brilliant II SYBR 1-step Master Mix reactions (Agilent, Santa Clara, CA, USA) following the manufacturer’s protocol. Samples were run in an Mx3005 qPCR cycler (Stratagene) with 240 nM of each primer, with or without RT, with cycling parameters as follows: 1 cycle at 50 °C for 30 min, 1 cycle at 95 °C for 10 min, 40 cycles of 95 °C for 30 s, 52 °C for 1 min and 72 °C for 45 s, and a dissociation curve cycle of 95 °C for 1 min, 52 °C for 30 s, and 95 °C for 30 s to confirm product specificity. If larger products were predicted, RNA samples were run with the Access RT-PCR kit (Promega, Madison, WI, USA) as per manufacturer’s protocol. Products were run on 1.5% agarose gels to visualize results. Primers targeted epitope sequences, and GFPuv (present on pRAM18dSGK) primers were used for quality control.

Protein from *R. parkeri* cultures was extracted using CelLytic B Cell Lysis Reagent (Sigma-Aldrich), then boiled for 4 min in 4× Laemmli buffer (BioRAD) before separation by SDS-PAGE on Any kD Mini PROTEAN precast gels (BioRAD). Proteins were transferred to a PVDF membrane and probed with antibodies against mKate (1:500; ThermoFisher monoclonal antibody TA180091) or GFPuv (1:1000; Novus Biologicals monoclonal antibody MAB4240; Centennial, CO, USA), then secondary antibodies (goat anti-mouse IgG HRP conjugate (ThermoFisher G-21040), 1:10,000). Protein detection was performed using a Pierce ECL kit (ThermoFisher).

### 2.8. Preparation of Rickettsiae for Inoculation of Mice

Wild-type and transformed mutant *R. parkeri* were grown in Vero cells at 34 °C and 4% CO_2_ in RPMI1640 (Gibco, ThermoFisher) supplemented with 10% FBS. Host cell-free rickettsiae were isolated from heavily infected Vero cells by vortexing with 60/90 rock tumbler grit (Lortone, Mukilteo, WA, USA), followed by passage through a 2 µm pore size filter to remove host cell debris, and centrifugation at 13,200× *g* for 5 min at 4 °C to pellet bacteria, as described previously [19]. Rickettsiae were then resuspended in RPMI1640 with 20% FBS and 10% DMSO, aliquoted into cryovials and stored in liquid nitrogen. To quantify the number of infectious bacteria per aliquot, cryovials were thawed in a 37 °C water bath, spun down, and the cell pellet resuspended in 0.6 mL fresh medium. A volume of 100 µL of rickettsiae were then added to replicate wells of a 24-well plate containing confluent Vero cells, centrifuged at 1500× *g* for 5 min and then incubated at 37 °C for 2 h. Wells were then washed twice with PBS to remove external rickettsiae, trypsin-treated to detach cells, and resuspended in 0.5 mL growth medium. Cells were spun down, and DNA was extracted using a Puregene kit (QIAGEN) following the manufacturer’s Gram-negative bacteria protocol. Quantification of *R. parkeri* in each sample was performed with qPCR targeting the *gltA* gene, as described above.

### 2.9. Mouse Challenge Experiments to Test Efficacy of Attenuated R. parkeri Mutant Expressing A. phagocytophilum Epitope Arrays

On day 0, twenty-one four-week-old female C3H/HeJ mice (Jackson Laboratory) were injected i.p. with 1 × 10^5^ *R. parkeri* G8::lox[virB9-mKate] in 200 μL PBS, and nine were inoculated with 200 μL PBS as controls. Mice were sorted into groups of three, and each group was weighed together daily throughout the experiment and monitored for signs of illness. Three days later, one group of vaccinated mice were euthanized and organs (heart, liver, lungs, spleen) were collected for determination of infection with the vaccine strain. On day 28, nine immunized mice were given a booster dose containing 1 × 10^5^ *R. parkeri* G8::lox[virB9-mKate] in 200 μL PBS. The other nine immunized mice and nine control mice were injected with 200 μL PBS. Seven days later (day 35), three mice from each group were sacrificed for collection of serum and organs for assessment of immune response to *R. parkeri* and *A. phagocytophilum,* and infection with the vaccine strain. Organs were stored at −20 °C for DNA extraction. On day 42, the remaining vaccinated and control mice were inoculated i.p. with either 1 × 10^6^ *R. parkeri* WT in 200 μL PBS (three mice/group) or 200 μL HL-60 culture infected (>50%) with *A. phagocytophilum* HGE1, containing approximately 1 × 10^5^ infected cells (three mice/group). Mice challenged with *R. parkeri* were euthanized three days post challenge (day 45), and serum and organs collected as above. Those challenged with *A. phagocytophilum* were euthanized seven days post challenge [30,45], for collection of serum and organs. About 35 µL of blood taken from these mice was transferred to flasks of approximately 1 × 10^6^ HL-60 to assess *A. phagocytophilum* infection by examination of Giemsa-stained cells and PCR using PER1/2 primers against the 16S RNA gene [20]. Spleen weights were measured from mice euthanized on days 35, 45, and 49.

### 2.10. Quantification of Bacterial Load in Mouse Tissues

*Rickettsia parkeri* were quantified using qPCR against the single copy *gltA* gene, as described above. *Anaplasma phagocytophilum* load was measured with a qPCR assay targeting the *msp5* gene [46] using 10-fold serial dilutions of an *msp5*-containing plasmid as a standard curve.

### 2.11. Immunofluorescence Assays

HL-60 cells infected with *A. phagocytophilum*, at a level of 80–90%, were resuspended in PBS and added to 18-well slides with approximately 2 × 10^3^ cells per well. Slides were air-dried, then fixed in acetone for 8 min [47]. Slides were blocked with FBS overnight at 4 °C in a humid chamber. Blocking agent was removed and pooled sera from mice, diluted at 1:50, 1:100, 1:200, 1:400, 1:800, and 1:1600 in PBST + 1% BSA, were added to slides in triplicate and incubated at RT for 1 h in a humid chamber. Slides were washed 3× in PBST, then secondary antibody (AlexaFluor488-conjugated goat anti-mouse, 1:1500 in PBST + 1% BSA) was added and incubated at RT for 1 h in a humid chamber. Slides were washed 3× in PBST, allowed to dry, and viewed by fluorescence microscopy on an Olympus BX61 confocal microscope at 40× magnification. IFAs against *R. parkeri* were performed with *R. parkeri-*infected ISE6 cells using a similar protocol.

### 2.12. Enzyme-Linked Immunosorbent Assays

Each recombinant epitope peptide array was diluted in freshly prepared 50 mM carbonate–bicarbonate buffer and used to coat a 96-well Nunc Immuno polysorb ELISA plate with 500 ng per well. Plates were blocked for 3 h with PBST + 5% BSA, then incubated 2 h with sera from recombinant protein-immunized mice at 1:100 dilution. Secondary antibody (goat anti-mouse IgG, AlexFluor488) was added at 1:2000 for 1 h. Fluorescence was measured at 485/528 on a BioTek Synergy H1 microplate reader, and readings were adjusted to the negative control (secondary antibody only). Wells were washed 3× with PBST between each step and all incubations were performed at RT. Each treatment was tested in triplicate wells.

To test reactivity of sera from mice vaccinated with the transformed *R. parkeri* G8::lox mutant, bacteria (*R. parkeri* WT, *R. parkeri* G8::lox[virB9-mKate], and *A. phagocytophilum*) were isolated from cell culture as described above, resuspended in 50 mM carbonate–bicarbonate buffer, and approximately 1 × 10^8^ bacteria in 100 µL were pipetted into wells of a 96-well Nunc Maxisorp Immuno ELISA plate (Thermo Scientific). The plate was incubated overnight at 4 °C, washed 3× with PBST, then blocked with PBST + 5% nonfat dry milk for 2 h at RT. After washing 3× in PBST, pooled mouse sera (1:100 in PBST + 1% milk) were added to each well for 1 h, followed by another wash 3× in PBST, and 1 h incubation with secondary antibody (HRP-conjugated goat anti-mouse, 1:10,000 in PBST + 1% milk). Wells were then washed 3× in PBST, and TMB substrate (Thermo Scientific 1-Step Ultra TMB-ELISA) was added to each well for 20 min, followed by addition of 2M hydrochloric acid to stop the reaction. Absorbance was read at 450 nm on a BioTek Synergy H1 microplate reader and readings were adjusted to a blank control containing no bacteria to which no sera were added. Each treatment was tested in triplicate wells, with uncoated wells and wells without serum added, acting as further negative controls.

### 2.13. Statistics

Statistical analyses were conducted in GraphPad Prism version 10. One-way ANOVA was used to compare pathogen loads or spleen weights between different mouse groups, whereas a two-way ANOVA was used to analyze *A. phagocytophilum* infection of cell cultures over time and by vaccination status. A *p*-value of <0.05 was considered statistically significant.

## 3. Results

### 3.1. Production of R. parkeri Transposon Mutants

A library of 80 *R. parkeri* mutants was generated using himar1 transposon mutagenesis for the random insertion of a himar1 transposon into TA dinucleotides in the *R. parkeri* genome (Table S2). The transposon was designed to be flanked with mismatched lox sequences for subsequent replacement using RMCE catalyzed by the Cre recombinase enzyme (Figure 1E), described in detail in the Methods Section. The library included 54 mutants with the transposon inserted intragenically and 26 with intergenic insertions. Twelve mutants were generated with the GFPuv/rif transposon, and the remaining sixty-eight mutants contained the mCherry/aadA transposon. Details of all generated transposon mutants are provided in Table S2.

### 3.2. Mouse Infectivity of Transposon Mutants

**Figure 2:**
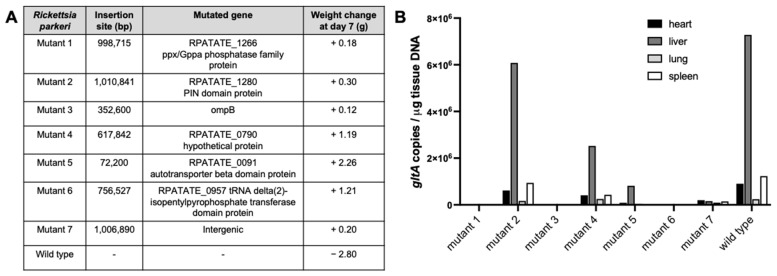
Comparison of murine infection with *Rickettsia parkeri* transposon mutants and wild type. (**A**) Details of disrupted genes in *R. parkeri* mutants and weight change in C3H/HeJ mice 7 days post infection (*n* = 2 mice/infection). Insertion site refers to position in *R. parkeri* Tate’s Hell genome. (**B**) Tissue load of *R. parkeri* mutants and wild type at day 7 as determined by qPCR quantification of *gltA.*

The results of WT *R. parkeri* dosage testing in mice indicated that the injection of 10^7^ or 10^6^ bacteria resulted in severe illness by day 3–4, requiring euthanasia. Mice in the other dosage groups initially gained weight but started to lose weight from days 3–4, indicating that infection had occurred and rickettsiae were replicating. Therefore, in initial experiments to test the infectivity of transposon mutants, a non-lethal dose of 10^3^ *R. parkeri* per mouse was used. Several mutants were found to be attenuated in infection when compared with WT *R. parkeri* in these preliminary in vivo experiments. The mutational analysis of gene function in rickettsiae is limited and has not always yielded the anticipated results [5,48]. Therefore, seven mutants were selected based on a range of expected phenotypes from insertions in genes predicted to be important for infection (such as *ompB*)*,* insertions in genes of unknown function, and intergenic insertions expected to have no effect. Infection with these seven *R. parkeri* mutants was assessed in C3H/HeJ mice at 7- and 14-days post infection in comparison with WT *R. parkeri*. None of the mutants induced weight loss in the infected mice, whereas mice infected with WT lost on average 2.8 g by day 7, equating to approximately 10% weight loss (Figure 2A). Of seven mutants tested, mutant 2 showed a similar rickettsial load in mouse tissues to those infected with WT *R. parkeri*, whilst mutants 4, 5, and 7 showed greatly reduced tissue loads, and *R. parkeri* were not detected in tissues for mutants 1, 3, and 6 (Figure 2B). By day 14, neither WT nor mutant *R. parkeri* were detected in mouse tissues.

### 3.3. Development of Anaplasma Epitope Arrays for Expression in R. parkeri

**Figure 3:**
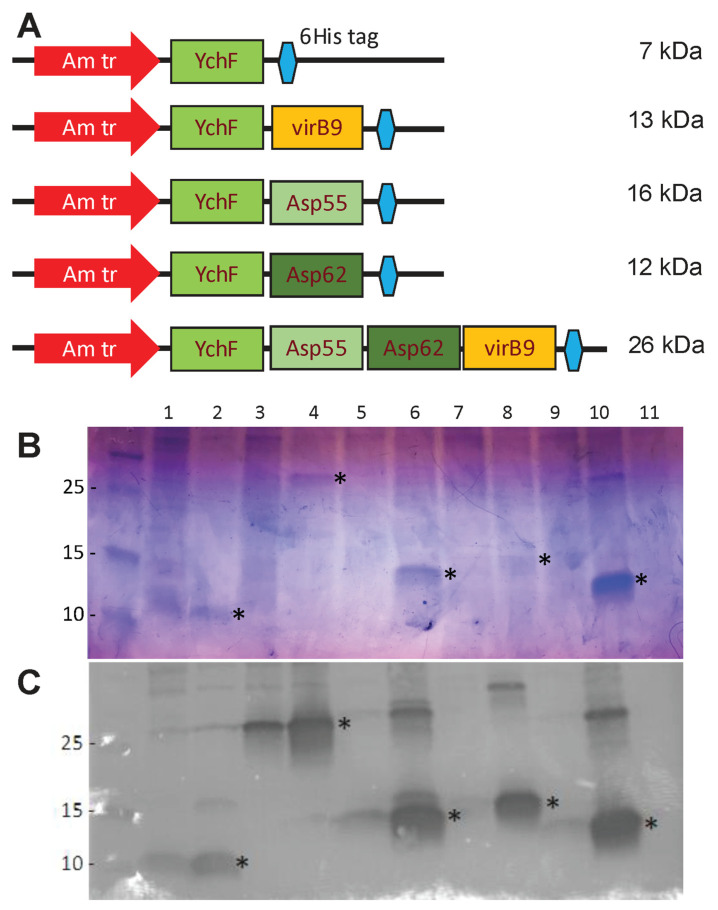
Expression of epitope arrays in *Escherichia coli.* (**A**) Diagram showing arrangement and expected molecular mass of different epitope arrays. (**B**,**C**) Expression of epitope arrays in *E. coli* BL21(DE3). Extracts from induced and uninduced cultures were separated by SDS-PAGE and stained with Coomassie (**B**) or probed with anti-6HisTag-HRP conjugate (**C**). 1. YchF uninduced; 2. YchF induced; 3. YchF-Asp55-Asp62-virB9 uninduced; 4. YchF-Asp55-Asp62-virB9 induced; 5. YchF-virB9 uninduced; 6. YchFvirB9 induced; 7. YchF-Asp55 uninduced; 8. YchF-Asp55 induced; 9. YchF-Asp62 uninduced; 10. YchF-Asp62 induced; 11. Untransformed BL21(DE3) control. Asterisks mark the expected band size for each array.

We aimed to employ the RMCE system to insert a cassette containing sequences of predicted *A. phagocytophilum* epitopes into an attenuated *R. parkeri* transposon mutant as a potential method for producing an attenuated vaccine strain protective against both rickettsiosis and anaplasmosis. Epitopes were selected bioinformatically from surface-exposed antigens of *A. phagocytophilum* that appear to be involved in host cell invasion [35], based on predicted linear B-cell epitopes and antigenicity (see Methods Section 2.4). Various configurations of the epitope array cassette were designed and first tested in *E. coli* and WT *R. parkeri* to confirm that they could be correctly expressed. Different configurations of the initial design of the epitope arrays were expressed in *E. coli* (Figure 3) and purified for the production of antibodies in mice. IFA showed that the serum of mice injected with all epitope arrays was reactive with *A. phagocytophilum* in HL-60 cells (Figure S1A). Mouse sera also recognized the epitope protein to which they had been immunized in a peptide ELISA, although due to the presence of YchF in all arrays, there was cross-reactivity of mouse sera to all epitope configuration peptides (Figure S1B).

**Figure 4:**
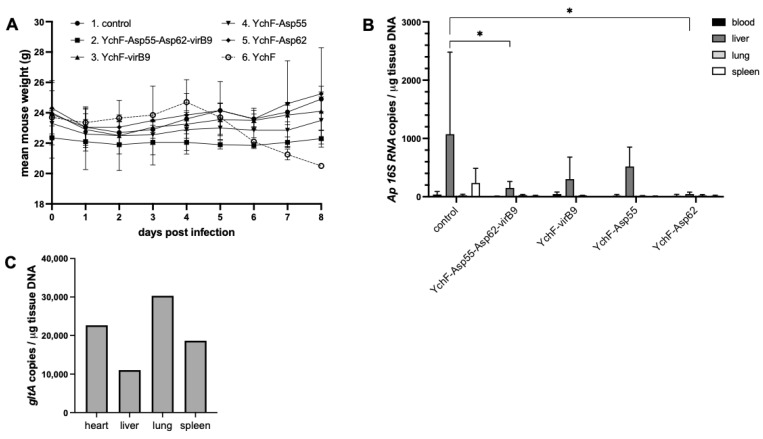
Challenge of epitope-immunized C3H/HeJ mice with *A. phagocytophilum* or *R. parkeri.* Mice (2 per group) were immunized with purified epitope protein produced in *E. coli.* Two injections of 50 μg were given 4 weeks apart, and pathogen challenge was performed 19 days after the booster dose. (**A**) Average weights of each mouse group following challenge with *A. phagocytophilum* (groups 1–5) or *R. parkeri* (group 6). (**B**,**C**) qPCR quantification of *A. phagocytophilum* (**B**) and *R. parkeri* (**C**) in tissues of challenged mice on day 8 post infection. Means were compared using a two-way ANOVA with Dunnett’s multiple comparisons test, * indicates *p* < 0.05.

Mice immunized with purified epitope protein showed similar weight gain compared with the negative control mice but exhibited reduced loads of *A. phagocytophilum* in tissues 8 dpi (Figure 4A,B), suggesting a protective effect of the epitope immunization, although due to the small number of animals used, this was only significant for *Anaplasma* loads in liver for two of the epitope combinations. In contrast, mice immunized with the YchF epitope alone showed no protection from *R. parkeri* challenge, with mice becoming severely ill (requiring euthanasia) within a week of infection (Figure 4A,C). This result may be explained by the fact that YchF plays a role in bacterial stress response pathways [49]; however, because a detailed investigation of this phenomenon was outside the scope of this project, we decided to delete YchF epitopes from future iterations of the arrays.

**Figure 5:**
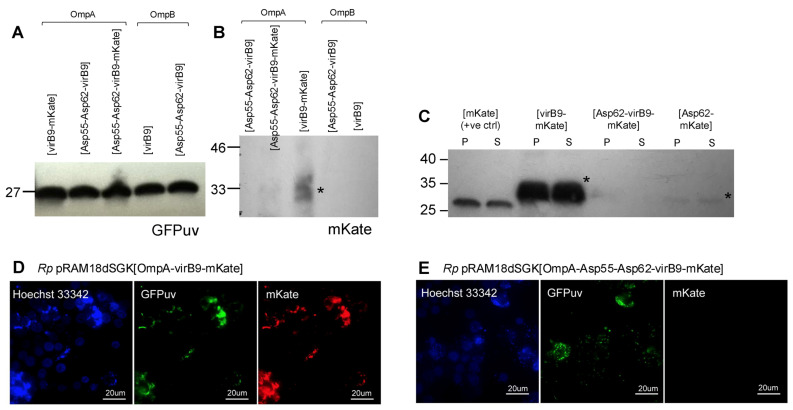
Detection of mKate-epitope fusion proteins expressed from pRAM18dSGK shuttle vectors in transformed WT *R. parkeri*. (**A**–**C**) Western blots of protein extracted from *R. parkeri* transformed with various iterations of epitope cassettes in pRAM18 shuttle vectors. Promoter OmpA or OmpB is shown above the constructs in A and B, whilst all transformants in C used the OmpA promoter. Western blotting against GFPuv confirmed successful transformation with shuttle vectors (**A**). mKate expression was detected in *R. parkeri* transformed with plasmids containing [virB9-mKate] and [Asp62-mKate], but not [Asp55-Asp62-virB9-mKate] or [Asp62-virB9-mKate] (**B**,**C**). Asterisks mark the expected size of mKate-epitope fusion proteins. Numbers on left of blots indicate protein size in kDa; expected size of GFPuv is 27 kDa, virB9-mKate and Asp62-mKate 33 kDa, Asp62-virB9-mKate 37 kDa, and Asp55-Asp62-virB9-mKate 46 kDa. In (**C**) P = pellet and S = supernatant, and the positive control is *R. parkeri* expressing mKate only from pRAM18dSFA. (**D**,**E**) Fluorescent microscopy of *R. parkeri*-infected ISE6 cells gave similar results with mKate visible in pRAM18dSGK[OmpA-virB9-mKate]-transformed rickettsiae (**D**) but not in those transformed with pRAM18dSGK[OmpA-Asp55-Asp62-virB9-mKate] (**E**).

Following successful expression in *E. coli*, epitope arrays were ligated into the pRAM18dSGK rickettsial shuttle vector [14] for expression in WT *R. parkeri.* Probing Western blots of protein extracts from transformed rickettsiae with anti-His-tag antibodies failed to detect the expression of epitope proteins at the expected sizes. Therefore, attempts were made to improve the chances of successful peptide array expression and translation by replacing the *Amtr* promoter with the native rickettsial *ompA* or *ompB* promoters. However, the expression of the epitope arrays was not detected by anti-His antibodies using either *omp* promoter, irrespective of growing rickettsiae in ISE6 or Vero cells. As a control, Western blot with anti-GFPuv antibodies confirmed the presence of GFPuv protein resulting from successful expression from the shuttle plasmid. Finally, epitope constructs were created that included an mKate fusion adjacent to the His-tag, allowing direct detection of expression of the epitope cassette in successfully transformed *R. parkeri.* In total, 32 variations of the cassette were constructed and inserted into the pRAM18dSGK shuttle plasmid; a complete list of all configurations is included in Table 2. Twenty-three of these were successfully transformed into *R. parkeri* and the presence of the correct size transcripts was observed for most constructs by RT-PCR, indicating successful expression in transformed rickettsiae (Table 2; Figure S2). The translation of mKate-epitope fusion protein and GFPuv was examined by Western blotting and fluorescent microscopy in a subset of these transformants (Figure 5). Whilst GFPuv was detected in all plasmid-transformed samples (Figure 5A), confirming the acquisition of the shuttle vector, mKate expression was only detected in *R. parkeri* transformed with shuttle vectors containing the virB9-mKate and Asp62-mKate sequences but not the Asp55-Asp62-virB9-mKate or Asp62-virB9-mKate sequences (Figure 5B,C). The presence/absence of red-fluorescent rickettsiae during the visualization of transformed *R. parkeri* by fluorescent microscopy confirmed these results (Figure 5D,E). Plasmid expression of the virB9 epitope appeared to be higher than that of the Asp62 epitope (Figure 5C).

**Figure 6:**
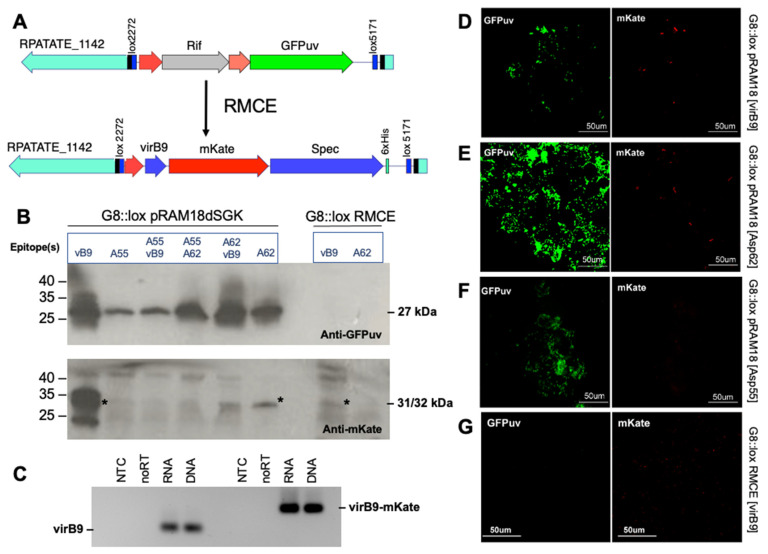
Detection of mKate-epitope fusion proteins in the *R. parkeri* G8::lox mutant. (**A**) Schematic showing recombinase-mediated cassette exchange in the G8::lox mutant. RMCE results in the replacement of the GFPuv/rif intragenic transposon insertion in RPATATE_1142 with a cassette containing the epitope, mKate, and spectinomycin/streptomycin (“Spec”) resistance sequences. Unlabeled red arrows indicate location of promoters (see Figure 1). (**B**) Western blot detection of GFPuv and mKate in G8::lox mutants transformed with pRAM18dSGK shuttle vectors containing mKate and epitope sequences and in G8::lox mutants that have undergone RMCE to replace GFPuv/rif transposon with mKate-epitope sequence. Abbreviations used for epitopes: A55 = Asp55; A62 = Asp62; vB9 = virB9. Asterisks mark the expected size of mKate-epitope fusion proteins. (**C**) RT-PCR of DNA/RNA extracts from *R. parkeri* G8::lox[virB9-mKate] with primers to virB9 and virB9-mKate. NTC = no template control; noRT = control without reverse transcriptase added; RNA = RNA with reverse transcriptase; DNA = positive control using DNA. (**D**–**F**) Fluorescent microscopy of ISE6 cells infected with *R. parkeri* G8::lox expressing GFPuv and mKate from pRAM18dSGK plasmid. mKate-epitope fusion protein was expressed in rickettsiae transformed with plasmids containing virB9-mKate (**D**) and Asp62-mKate (**E**) sequences but not in those containing Asp55-mKate sequences (**F**). (**G**) Fluorescent microscopy of *R. parkeri* G8::lox mutant after RMCE, showing replacement of GFPuv-containing insertion with virB9-mKate cassette.

Due to the addition of the mKate reporter to the epitope arrays, we were constrained to using transposon mutants with a GFPuv/rif transposon insertion for subsequent RMCE introducing epitope arrays. A transposon mutant (designated *R. parkeri* G8::lox) with an insertion into the RPATATE_1142 gene encoding an N-acetylmuramoyl-L-alanine amidase family protein was selected for further genetic modification using RMCE to incorporate epitope arrays (Figure 6A). Based on the successful expression of virB9-mKate and Asp62-mKate from shuttle vectors in WT *R. parkeri*, the *R. parkeri* G8::lox transposon mutant was transformed with pRAM18dSGK shuttle vectors containing different configurations of epitopes fused to mKate. Similar to in WT, the expression of epitope-mKate fusion protein was detected only in the *R. parkeri* G8::lox transformants expressing virB9-mKate and Asp62-mKate from the plasmid by both Western blotting and fluorescent microscopy (Figure 6).

The G8::lox transposon mutant was separately transformed with RMCE plasmids (Figure 1) to replace the mutant’s intragenic GFPuv/rif sequence resulting from genome insertion mutagenesis with either the virB9-mKate or the Asp62-mKate cassette (Figure 6A). Western blot analysis detected mKate expression in *R. parkeri* G8::lox with the virB9-mKate insertion but not with the Asp62-mKate insertion (Figure 6B). GFPuv was detected in all transformants expressing the pRAM18dGSK plasmid but not in the two RMCE mutants, indicating a successful replacement of the loxHimar insertion containing GFPuv with the epitope-mKate-containing cassette (Figure 6B). The expression of virB9-mKate following RMCE in *R. parkeri* G8::lox[virB9-mKate] was also confirmed by RT-PCR (Figure 6C). The Western blot results were consistent with fluorescence microscopy findings for each transformant (Figure 6D–G).

### 3.4. Testing Immunogenicity of Epitope Arrays In Vivo

**Figure 7:**
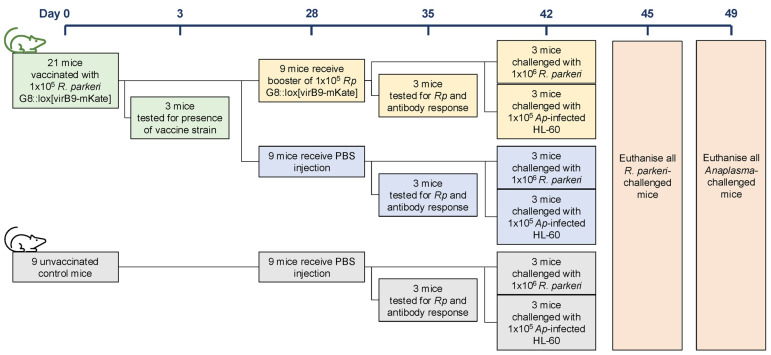
Outline of mouse vaccination-challenge experiment.

We reasoned that an attenuated *R. parkeri* transposon mutant expressing *A. phagocytophilum* epitopes could be used as a live-attenuated vaccine to induce broad protective immunity against SFG rickettsiae and *A. phagocytophilum* in mice. Thus, we immunized mice with *R. parkeri* G8::lox[virB9-mKate] and challenged animals with either WT *R. parkeri* or *A. phagocytophilum*. Different groups of mice received either a single inoculation of 1 × 10^5^ *R. parkeri* G8::lox[virB9-mKate] at day 0 (“vaccinated” group), or the same treatment at day 0 followed by a booster dose containing the same number of *R. parkeri* G8::lox[virB9-mKate] at day 28 (“boosted” group; Figure 7). A control group received PBS injections at days 0 and 28. On day 35, three mice from each group were euthanized to obtain serum for antibody investigation and tissue samples to determine the persistence of *R. parkeri* G8::lox[virB9-mKate]. On day 42, all mice were challenged with either 1 × 10^6^ WT *R. parkeri* or 1 × 10^5^ HL60 cells infected with *A. phagocytophilum*. The *R. parkeri*-challenged mice were euthanized three days later whilst *A. phagocytophilum*-challenged mice were euthanized seven days later, to take into account the different courses of infection of each pathogen (Figure 7).

**Figure 8:**
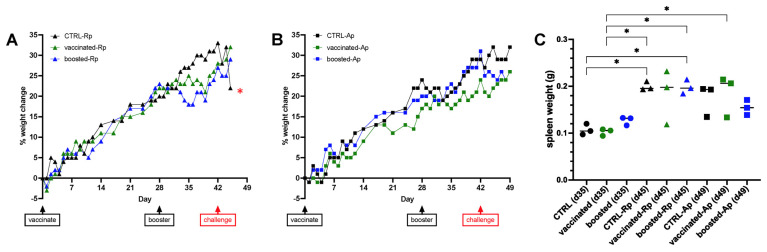
Mouse responses to pathogen challenge following vaccination with *R. parkeri* G8::lox[virB9-mKate]. (**A**,**B**) Weights of C3H/HeJ mice challenged with (**A**) *R. parkeri* or (**B**) *A. phagocytophilum*. (**C**) Spleen weights of challenged mice. Means were compared using a one-way ANOVA with Tukey’s multiple comparisons test, * indicates *p* < 0.05. Vaccination status is denoted by color (black = unvaccinated control; green = vaccinated; blue = boosted) and pathogen challenge is shown by shape (circle = unchallenged control; triangle = *R. parkeri*; square = *A. phagocytophilum*).

All mouse groups gradually gained weight after initial vaccination (Figure 8A,B). Following the day 28 booster dose or PBS injection, there was a slight drop or levelling off of weight gain in multiple groups, although mice appeared to recover. Following challenge with WT *R. parkeri*, the unvaccinated control group appeared visibly ill, exhibiting ruffled fur and hunched postures, and showed a dramatic weight loss of approximately 10%, whereas the vaccinated and boosted groups displayed no weight loss or other conditions indicative of disease (Figure 8A). As expected, no signs of illness were observed in mice challenged with *A. phagocytophilum*, although the boosted group showed a slight loss of weight (Figure 8B). There were no differences in spleen weight between control, vaccinated, and boosted groups of mice at days 35, 45, or 49 (Figure 8C); the only significant differences observed in spleen weight corresponded to time, i.e., before and after challenge (day 35 vs. day 45 or 49).

**Figure 9:**
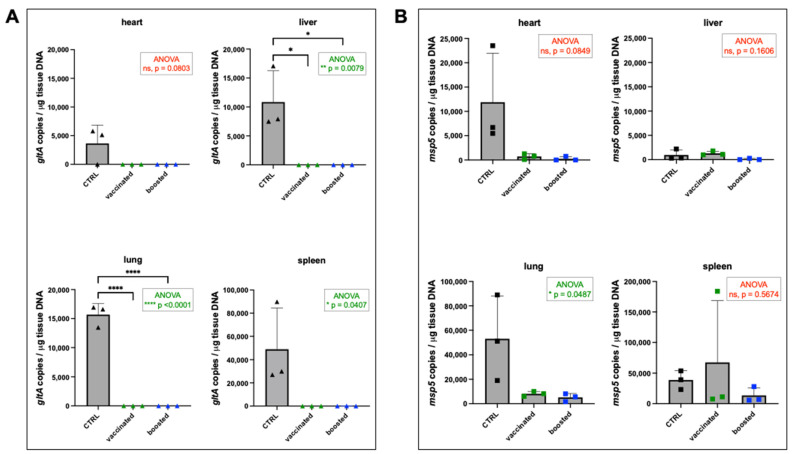
Tissue load of pathogens in challenged mice vaccinated with *R. parkeri* G8::lox[virB9-mKate]. Copy numbers of (**A**) *R. parkeri* and (**B**) *A. phagocytophilum* in tissues of challenged mice. Means were compared using a one-way ANOVA with Tukey’s multiple comparisons test: ns = not significant; * *p* < 0.05; ** *p* < 0.01; **** *p* <0.0001.

**Figure 10:**
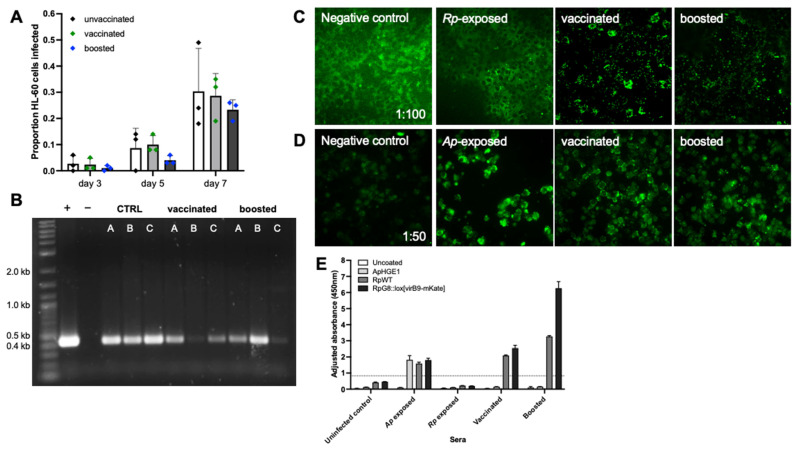
Analysis of response to infection with *A. phagocytophilum* and *R. parkeri* in mice vaccinated with *R. parkeri* G8::lox[virB9-mKate]. (**A**) Giemsa results from blood cultures of *A. phagocytophilum*-challenged mice. Data were analyzed by two-way ANOVA: No significant difference by vaccination status, *p* < 0.0001 by time. (**B**) PER1/2 PCR results from day 11 blood cultures of *A. phagocytophilum*-challenged mice. Expected band size is 451 bp. A, B, and C are individual mice from each group. (**C**) IFA on *R. parkeri*-infected ISE6 cells using sera from unvaccinated, unchallenged mice (negative control), sera taken from mice 3 dpi with WT *R. parkeri* (*Rp*-exposed), and sera from vaccinated and boosted mice (taken on day 35). 40× magnification. (**D**) IFA on *A. phagocytophilum*-infected HL60 cells using sera from unvaccinated, unchallenged mice (negative control), sera taken from mice 7 dpi with WT *A. phagocytophilum* (*Ap*-exposed), and sera from vaccinated and boosted mice (taken on day 35). 40× magnification. (**E**) ELISA against wells coated with *A. phagocytophilum*, WT *R. parkeri*, or *R. parkeri* G8::lox[virB9-mKate] and probed with serum from five groups of mice (listed on x-axis). Well contents are indicated by color of bars. Dotted line represents the positive cut-off value calculated as mean + 3 standard deviations of the negative control. Absorbance was adjusted to a blank well containing no bacteria to which no sera were added.

Rickettsiae were not detected in tissues of mice that had been vaccinated or boosted (Figure 9A), which was significantly different from control mice for liver (*p* = 0.0079), lung (*p* < 0.0001), and spleen (*p* = 0.0407) but not for heart (*p* = 0.0803). In contrast, vaccinated and boosted mice appeared to have little immunological protection from challenge with *A. phagocytophilum* (Figure 9B); *Anaplasma* loads were similar across groups in liver (*p* = 0.1606) and spleen (*p* = 0.5674), and although they were higher in heart tissues of control mice, this was not significant (*p* = 0.0849). However, *Anaplasma* loads in the lung was slightly higher in the control mice (*p* = 0.0487) than in the vaccinated or boosted groups (Figure 9B). Furthermore, blood cultures prepared from *A. phagocytophilum*-challenged mice showed no significant differences based on vaccination status, with infection progressing similarly in cultures prepared from control, vaccinated, and boosted mice (Figure 10A). The presence of *A. phagocytophilum* DNA was confirmed in all blood cultures by PCR (Figure 10B).

The analysis of mouse tissues for the persistence of *R. parkeri* G8::lox[virB9-mKate] at day 3 after initial vaccination showed that numbers of rickettsiae were very low, indicative of an attenuated infection phenotype, whilst at day 35 (7 days after booster) and day 49, rickettsiae were undetectable or extremely low (Figure S3).

IFA using serum from vaccinated and boosted mice taken at day 35 showed a strong labeling of WT *R. parkeri* in ISE6 cells at all dilutions tested (Figure 10C), indicating a significant antibody response, and was stronger than that of serum from control mice taken at day 45 (3 dpi after infection with WT *R. parkeri* (Figure 7)). This difference may be due to the insufficient time for the control mice to develop a strong antibody response. Sera from vaccinated and boosted mice also labelled *A. phagocytophilum* morulae within infected HL-60 cells at dilutions of up to 1:200 (Figure 10D). ELISA comparing sera from vaccinated and boosted mice tested in wells containing either *A. phagocytophilum*, WT *R. parkeri*, or *R. parkeri* G8::lox[virB9-mKate] showed binding to *R. parkeri*-coated wells but not to *A. phagocytophilum*-coated wells (Figure 10E). Interestingly, sera from *A. phagocytophilum*-infected mice showed reactivity to *R. parkeri*-coated wells, as well as to *A. phagocytophilum*-containing wells. Sera from vaccinated and boosted mice bound more strongly to wells coated with *R. parkeri* G8::lox[virB9-mKate] than to those coated with WT *R. parkeri*.

## 4. Discussion

The obligate intracellular nature of the Rickettsiales has made the genetic manipulation of these bacteria extremely difficult until relatively recently [50], which has been a major obstacle to investigating gene function. The application of transposon mutagenesis to *A. phagocytophilum* [21] and *R. prowazekii* [4,24] paved the way for the development of this tool for use in other pathogenic Rickettsiales including *A. marginale* [51], *Ehrlichia chaffeensis* [22], and *R. parkeri* [10,52]*,* although it remains a challenging approach. The development of shuttle vectors allowing the stable transformation of genes of interest into diverse *Rickettsia* species [14] enabled the complementation of genes knocked out by transposon mutagenesis in *R. parkeri* [10]. Using these genetic tools, rickettsiologists have begun to uncover the function of genes required for pathogenesis, and the recent implementation of conditional gene expression and CRISPR interference-mediated knockdown in *R. parkeri* [53] and *R. rickettsii* [54] will further accelerate discovery. Here, we have further modified the transposon mutagenesis approach to allow the exchange of sequences carried within the transposon with sequences of interest, which may be used for the complementation of the mutagenized gene, the expression of non-native sequences, or the analysis of non-coding RNAs and regulatory elements. Advantages of this system include the ability to introduce larger sequences than can be inserted by transposon mutagenesis and the stable intragenic expression of the inserted gene of interest.

Transposon mutagenesis of certain *R. parkeri* genes results in an attenuated infection phenotype [6,10,55]; this has been attributed to the roles of specific gene products in cell–cell spread [10] or the evasion of host immune responses [55]. We identified a range of additional *R. parkeri* genes whose mutation resulted in reduced in vivo infectivity and should be investigated further to provide insights into the roles that these genes play in rickettsial pathogenesis. The G8::lox mutant employed in this study contained a transposon insertion in the RPATATE_1142 gene which encodes an N-acetylmuramoyl-L-alanine amidase family protein. These enzymes are involved in cell wall breakdown and recycling, and therefore defects in bacterial cell wall integrity may have been involved in the attenuated mouse infection observed with this mutant; further investigation will be required to elucidate the function of this protein and its potential roles in *R. parkeri* biology. Although this mutant was not included in our preliminary analysis of mouse infectivity of transposon mutants, the low number of the G8::lox[virB9-mKate] mutant rickettsiae detected (only a few hundred copies compared with thousands of copies for a WT infection) in mice 3 dpi in the vaccination study indicated that it had reduced virulence in vivo. Moreover, the incorporation of GFPuv and rifampicin resistance on the transposon facilitated the detection and selection of mutants following successful RMCE to swap in the modified epitope arrays incorporating *mKate* and *aadA* codons.

One application of the RMCE approach, which we began to investigate in this study, is the introduction of epitope sequences from various tick-borne pathogens into the introduced cassette, turning an attenuated *R. parkeri* mutant into a multivalent live-attenuated vaccine that would confer immunity to SFG rickettsiae as well as the pathogens covered by the epitopes. By inserting an array of epitopes from multiple tick-borne pathogens in this way, a vaccine protective against a range of tick-borne diseases could be developed. Our collection of attenuated mutants represents a valuable pool for future vaccine studies when complemented using RMCE to express various epitopes from tick-borne pathogens. To test this approach, we incorporated epitope sequences from *A. phagocytophilum* proteins that have been identified as promising vaccine candidates. The *Anaplasma* surface proteins Asp55 and Asp62 were recognized by serum from an anaplasmosis patient, and anti-Asp55 and anti-Asp62 sera reduced infection of HL60 cells by *A. phagocytophilum* [35]. VirB9-1 is a component of the T4SS that has shown promise as a vaccine candidate against *Anaplasma marginale* infection in cattle [34,37]. However, cattle vaccinated with recombinant virB9-1, virB9-2, virB10, and virB11 were not protected against challenge with virulent *A. marginale*, despite mounting a specific antibody response to the antigens [56]. Similarly, although sera from mice vaccinated with recombinant virB9-1 and virB9-2 recognized corresponding proteins in *A. phagocytophilum*, the immunization was ineffective at preventing *A. phagocytophilum* infection in mice [57]. Likewise, we found that mice vaccinated with *R. parkeri* expressing a virB9-1 epitope failed to develop protective immunity against subsequent *A. phagocytophilum* infection, even though they generated antibodies that bound the pathogen. Further experimentation using different antigens and epitopes will be required to perfect this approach and determine whether it is feasible for generating a novel vaccine conferring broad protectivity against multiple tick-borne pathogens. It is appreciated that vaccines consisting of epitopes from multiple antigens confer better protection than vaccines based on a single antigen [58,59]. Although our original plan was to include multiple antigenic epitopes, the expression of these arrays was difficult to achieve in *R. parkeri*, despite being successful in *E. coli.* Only virB9 and Asp62 were successfully expressed as mKate fusion proteins in *R. parkeri,* with differing strengths of expression. Similarly, the expression of mKate-epitope fusion proteins appeared to be low relative to GFPuv expression, which was transcribed from the same shuttle vector plasmid (see Figure 6). Possibly, the expression of two fluorophores is energetically too demanding, as metabolic resources must be diverted to their production, or too toxic for the rickettsiae to be able to sustain. It has been shown that the production of fluorescent proteins induces H_2_O_2_ that can damage cells and bacteria [60,61]. We also noticed that the expression of mKate in these plasmid-transformed rickettsiae reduced over time, being lower in higher sub-passage *R. parkeri* transformants. Further modifications to the shuttle vector system to improve its stability, e.g., by using a less strong promoter, or its ability to incorporate large inserts could be attempted in the future. It is unclear why some epitopes or epitope combinations were not successfully expressed by transformed *R. parkeri.* The optimization of epitopes, for example codon optimization to improve expression, may be required for them to be expressed successfully in this pathogen. However, codon usage in AT-rich bacteria including the Rickettsiales is similar, favoring U or A in the first and third positions [62].

Although we did not further investigate the effects of epitope arrays containing the YchF epitope, the negative outcomes were unexpected. The YchF sequences (from a gene encoding a ribosome-binding ATPase) were included in the hope of enhancing the expression of arrays, but its puzzling effect on mouse mortality prompted us to search the literature for a possible explanation. Considering the body weight data showing that YchF-immunized mice did not recover following challenge (Figure 4A), we concluded that immunization against rickettsial YchF proved deleterious for mice. Epitopes from this protein were included because of its presumptive role as an ATPase that binds to both the 70S ribosome and the 50S ribosomal subunit. However, research indicates that it is a highly conserved negative regulator of the oxidative stress response in both bacteria and eukaryotes [49,63], which is an important pathogenicity mechanism of rickettsiae. Whilst the overexpression of YchF has been shown to reduce resistance to stress in *E. coli*, plants, and human cells [49,63,64], the deletion of the gene in the bacteria *Vibrio vulnificus* and *Streptococcus pneumoniae* reduced virulence in mice [65,66]. In *V. vulnificus*, deleting *ychF* was linked to the reduced transcription of the virulence factor RtxA1 toxin [65]; therefore, the overexpression of a portion of YchF in *R. parkeri* could potentially increase its mouse infectivity through an increased transcription of virulence factors. However, since the portion of YchF used as the epitope in this study did not contain its catalytic site, this seems unlikely. An alternative explanation, which might arise due to the highly conserved nature of this protein across bacteria and eukaryotes [67], is that the epitope induced auto-immunity against the murine YchF homolog, which may cause increased mortality. Further research into the roles of YchF in *R. parkeri* virulence would be interesting and could shed light on our observed results.

Building on previous successes in transposon mutagenesis, this research has developed a genetic tool for the exchange of the inserted transposon with a cassette containing genes of interest, which can be applied to the study of gene function in rickettsiae. The potential for the use of this method in other areas, such as the analysis of gene function, non-coding RNAs, promoters, and regulatory elements, will also benefit the field. Although our attempts to apply this method to the generation of a novel attenuated vaccine were unsuccessful, this remains a promising use of this technology that could be further explored after the identification of effective antigens and optimization of their epitopes for expression in *R. parkeri.*

## Figures and Tables

**Table 1:** Epitopes selected based on predicted characteristics of immunogenicity, surface accessibility, beta-turn presence, and hydrophilicity.

Antigen	Amino Acid Residues	Predicted Characteristics	Amino Acid Sequence
Asp55	467–528	antigenic, surface, hydrophilic	VDGENTLKDLVVGVGYNLFSKGSTSLEVFLNCHMFSVQHKFNIHEYKVSTESKVSTESKVYT
Asp62	111–148	antigenic, beta-turn	EYLSDSGTAYGADFQVMVPEVNSAVEVGKAFINRGSRA
virB9-1	98–145	antigenic, beta-turn	EKEGHTNMLIETSKGRSYAFDLISTAIPLSGGAASSINKLGKTNSALA
YchF	134–221	antigenic, surface-accessible	KVLGEDKPARVLNEALRVDNLKQLQLITSKPVLYICNVLEKDAAIGNEFTK

**Table 2:** List of constructed epitope arrays.

Construct	Successfully Transformed into *R. parkeri*	Expression Confirmed by RT-PCR
Amtr promoter-[YchF-Asp55-Asp62-VirB9]-6His Tag	Yes	partly
Amtr Promoter-[YchF]-6His Tag	No	-
Amtr Promoter-[YchF-Asp62]-6His Tag	Yes	Yes
Amtr Promoter-[YchF-Asp55]-6His Tag	Yes	Yes
Amtr Promoter-[YchF-VirB9]-6His Tag	No	-
Amtr Promoter-[Asp55-Asp62-VirB9]-6His Tag-OmpA terminator	No	-
Amtr promoter-[VirB9]-6His Tag-OmpA terminator	No	-
OmpA Promoter-[YchF-Asp55-Asp62-VirB9]-6His Tag-OmpA terminator	Yes	Yes
OmpA Promoter-[YchF]-6His Tag-OmpA terminator	Yes	Yes
OmpA Promoter-[YchF-Asp62]-6His Tag-OmpA terminator	Yes	Yes
OmpA Promoter-[YchF-Asp55]-6His Tag-OmpA terminator	No	-
OmpA Promoter-[YchF-VirB9]-6His Tag-OmpA terminator	Yes	Yes
OmpA Promoter-[Asp55-Asp62-VirB9-mKate]-6His Tag-OmpA terminator	Yes	Yes
OmpA Promoter-[VirB9-mKate]-6His Tag-OmpA terminator	Yes	Yes
OmpA Promoter-[Asp55-Asp62-VirB9]-6His Tag-OmpA terminator	Yes	Yes
OmpA Promoter-[VirB9]-6His Tag-OmpA terminator	Yes	Yes
OmpB Promoter-[Asp55-Asp62-VirB9]-6His Tag-OmpA terminator	Yes	Yes
OmpB Promoter-[VirB9]-6His Tag-OmpA terminator	Yes	Yes
OmpB Promoter-[YchF-Asp55-Asp62-VirB9]-6His Tag-OmpA terminator	No	-
OmpB Promoter-[YchF]-6His Tag-OmpA terminator	No	-
OmpB Promoter-[YchF-Asp62]-6His Tag-OmpA terminator	Yes	Yes
OmpB Promoter-[YchF-Asp55]-6His Tag-OmpA terminator	No	-
OmpB Promoter-[YchF-VirB9]-6His Tag-OmpA terminator	Yes	Yes
OmpA Promoter-[virB9-mKate]-6His Tag-OmpA terminator	Yes	Yes
OmpA Promoter-[Asp55-mKate]-6His Tag-OmpA terminator	Yes	No
OmpA Promoter-[Asp55-Asp62-VirB9-mKate]-6His Tag-OmpA terminator	No	-
OmpA Promoter-[Asp55-virB9-mKate]-6His Tag-OmpA terminator	Yes	No
OmpA Promoter-[Asp55-Asp62-mKate]-6His Tag-OmpA terminator	Yes	Partly
OmpA Promoter-[Asp62-virB9-mKate]-6His Tag-OmpA terminator	Yes	Partly
OmpA Promoter-[Asp62-mKate]-6His Tag-OmpA terminator	Yes	Yes
OmpA Promoter-6His Tag-[Asp62-mKate]-OmpA terminator	Yes	Yes
OmpA Promoter-[Asp62-mKate]-8His Tag-OmpA terminator	Yes	Yes

## Data Availability

The original contributions presented in this study are included in the article/Supplementary Materials. Further inquiries can be directed to the corresponding author(s).

## Supplementary Materials

The following supporting information can be downloaded at: https://www.mdpi.com/article/10.3390/vaccines13020109/s1, Figure S1: Reactivity of epitope-immunized mouse sera to *A. phagocytophilum* and recombinant peptides; Figure S2: Transcription of epitope arrays in plasmid-transformed *R. parkeri*; Figure S3: Persistence of vaccine strain *R. parkeri* G8::lox[virB9-mKate] in mice; Table S1: List of primers used in the study; Table S2: List of transposon mutants generated; Table S3: Densitometry data from Western blots.
