# Supplementary material for: Development of a Recombinase-Mediated Cassette Exchange System for Gene Knockout and Expression of Non-Native Gene Sequences in Rickettsia
Source: Vaccines (Basel). 2025 Jan 22;13(2):109. doi: 10.3390/vaccines13020109 (PMC11861799; doi:10.3390/vaccines13020109)

Original gels and blots showing expression of epitope arrays in *E. coli* and *R. parkeri*. (A) Coomassie gel and (B) Western blot on *E. coli* protein extracts, shown in Figure 3. (C) Original GFPuv and (D, E) mKate Western blots on *R. parkeri,* shown in Figure 5A-C. Protein gels used PageRuler Prestained Protein ladder (Thermo Scientific)

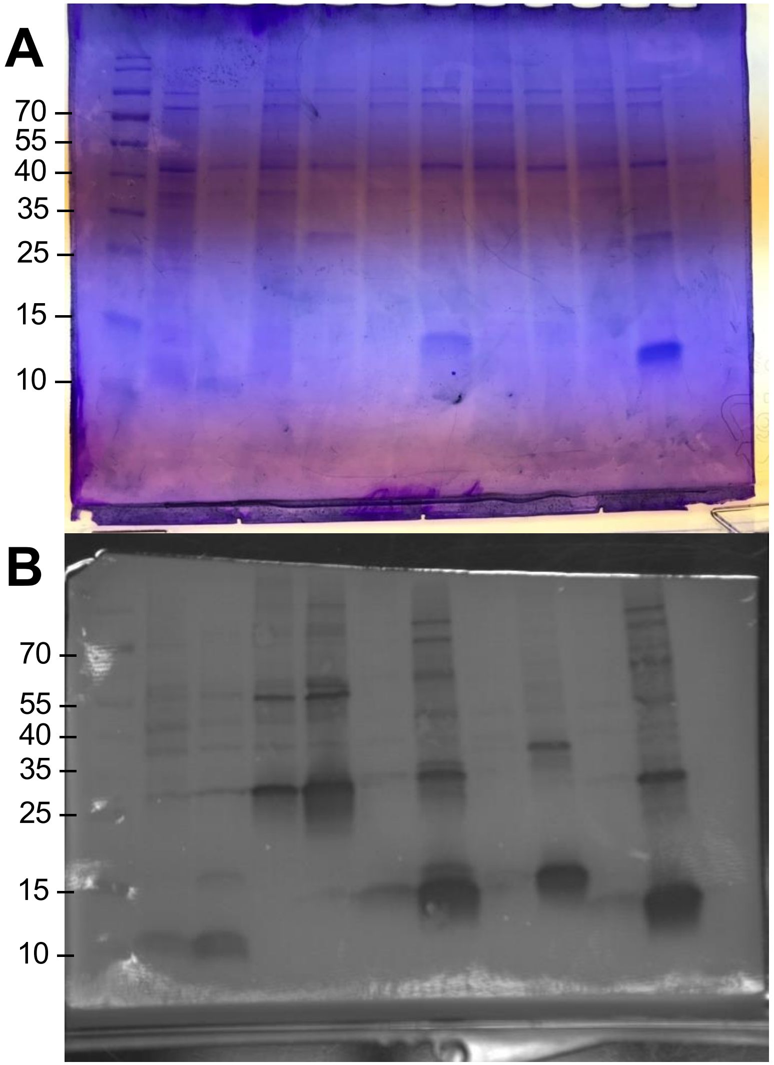

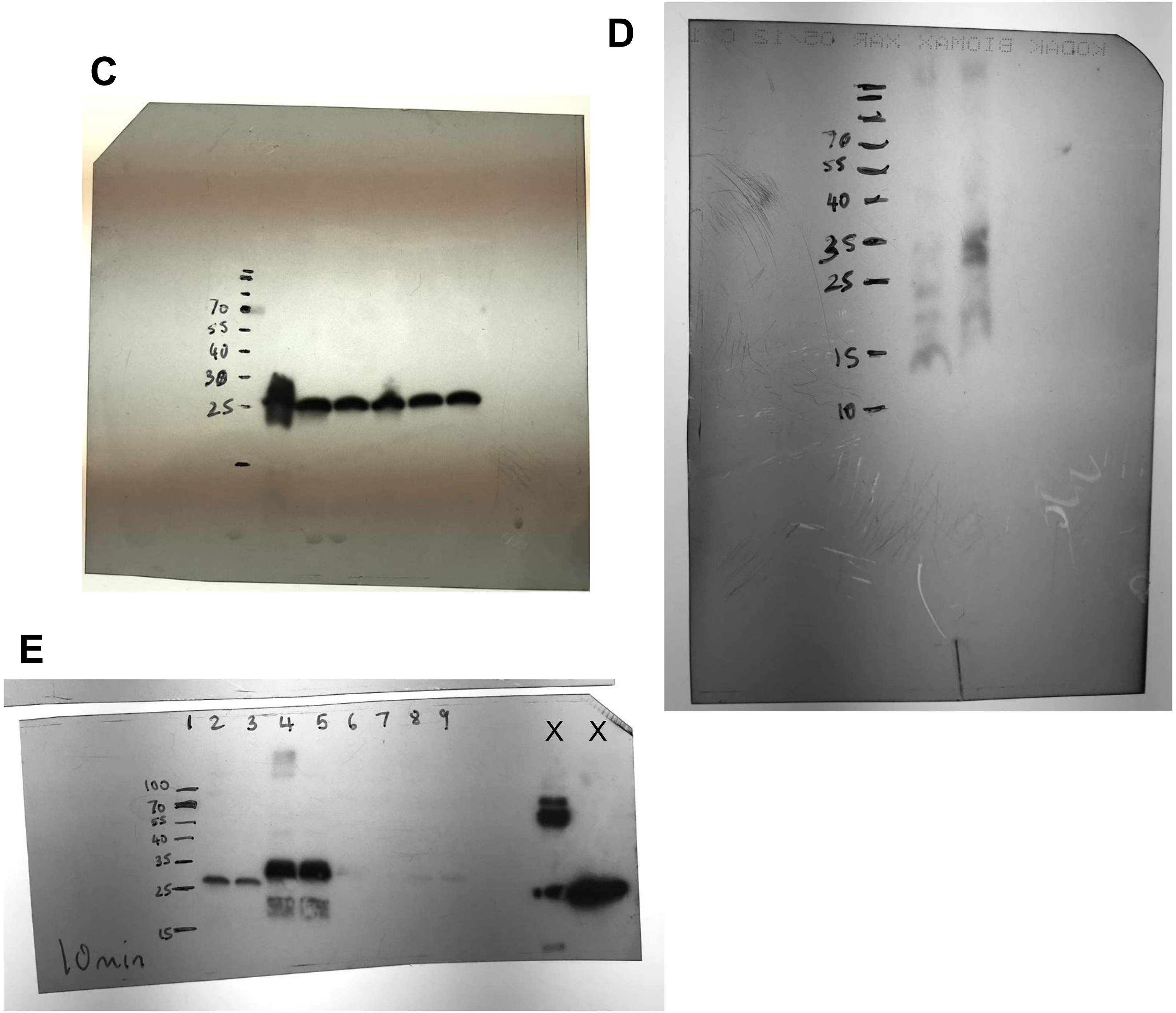

Original gels and blots showing expression of epitope arrays in *E. coli* and *R. parkeri*. (F) Original GFPuv and (G) mKate Western blots shown in Figure 6B. (H) Gel of RT-PCR analysis that demonstrate transcription of epitope arrays in *R. parkeri*, shown in Figure 6C. Protein gels used PageRuler Prestained Protein ladder (Thermo Scientific) and DNA gels used 1kb Plus DNA ladder (New England Biolabs).

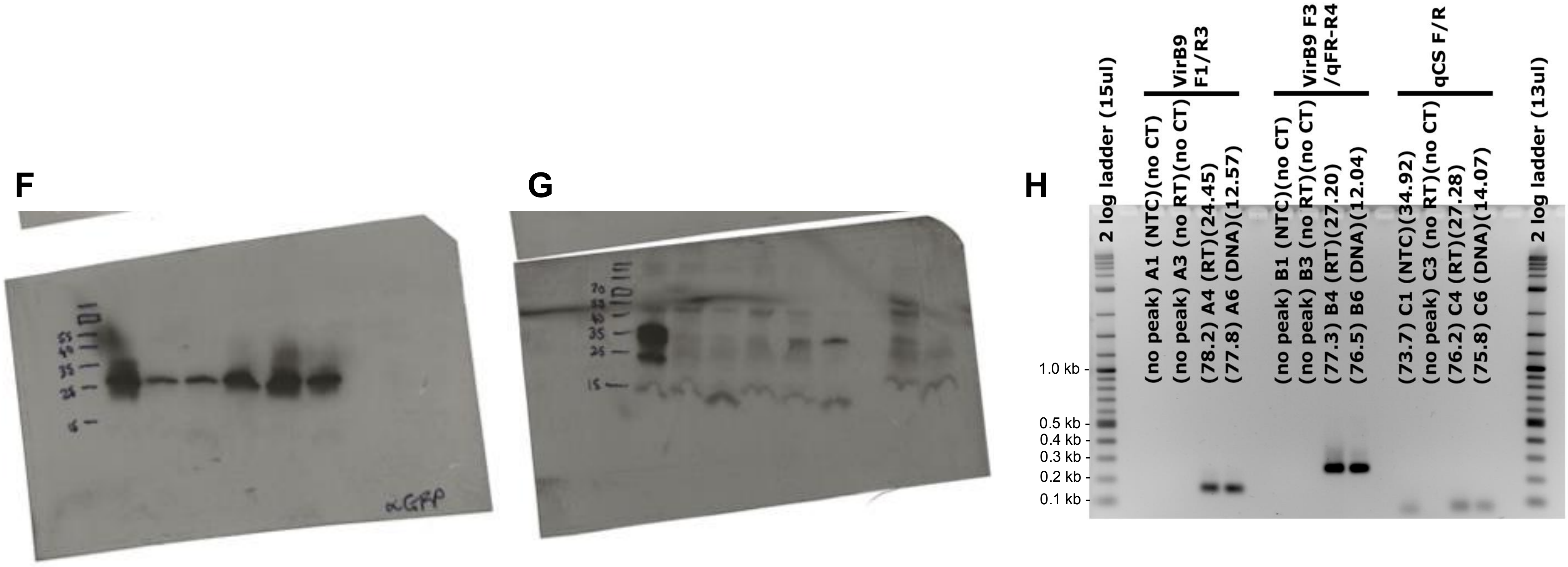

Supplement: Supplementary file 1 [file vaccines-13-00109-s001.zip › Original blot and gel images.pdf]
