# Supplementary material for: Development of a Recombinase-Mediated Cassette Exchange System for Gene Knockout and Expression of Non-Native Gene Sequences in Rickettsia
Source: Vaccines (Basel). 2025 Jan 22;13(2):109. doi: 10.3390/vaccines13020109 (PMC11861799; doi:10.3390/vaccines13020109)

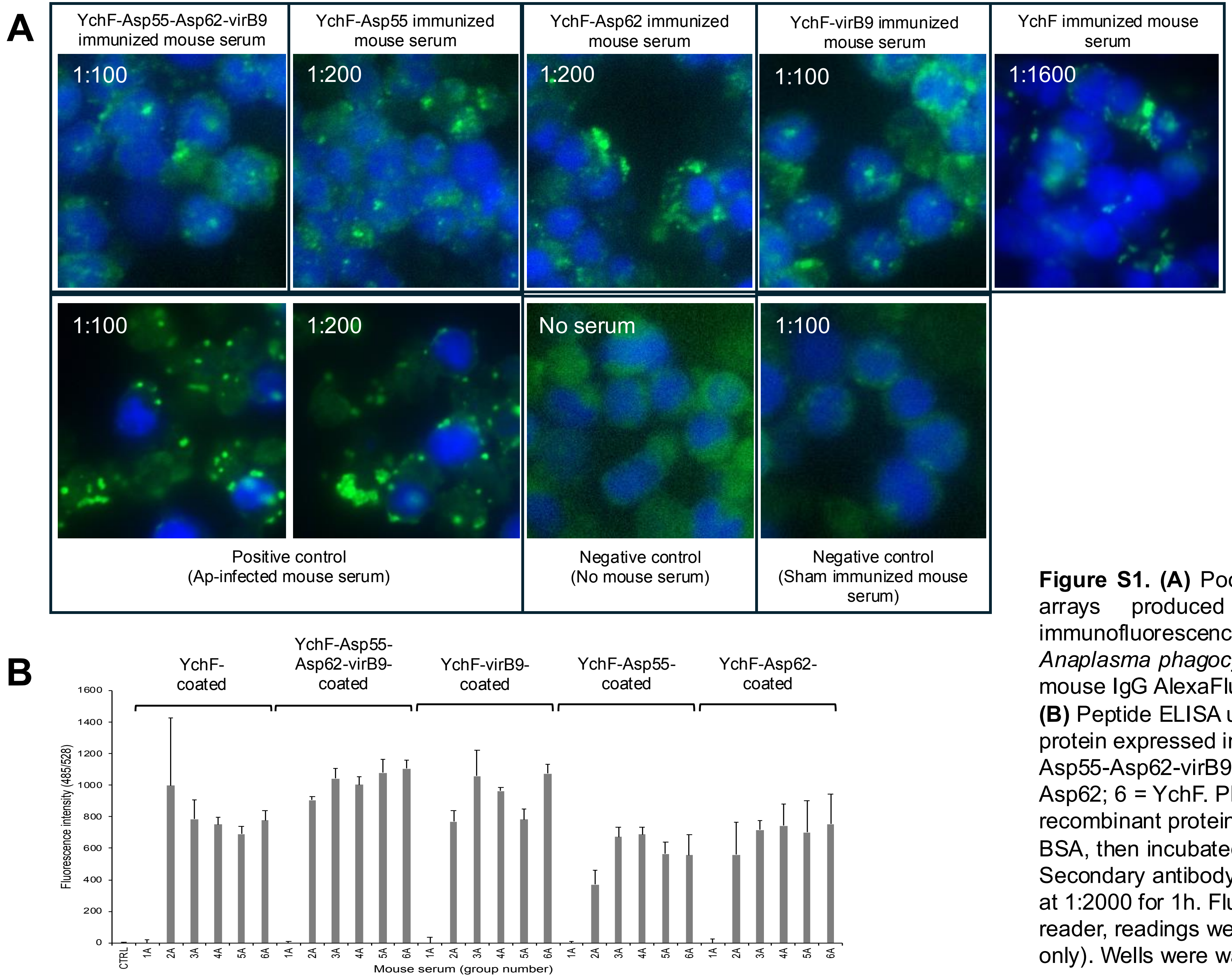

Figure S1. (A) Pooled sera from mice injected with purified epitope arrays produced in *E. coli* BL21(DE3) were used in immunofluorescence assays against HL-60 cells infected with *Anaplasma phagocytophilum*. The secondary antibody was goat anti-mouse IgG AlexaFluor488 conjugate used at 1:1000.

(B) Peptide ELISA using sera from mice immunized with recombinant protein expressed in *E. coli*. 1 = control (elution buffer only); 2 = YchF-Asp55-Asp62-virB9; 3 = YchF-virB9; 4 = YchF-Asp55; 5 = YchF-Asp62; 6 = YchF. Plate was coated with 500ng/well of each recombinant protein epitope. Plates were blocked 3h with PBST + 5% BSA, then incubated 2h with sera from mice at 1:100 dilution. Secondary antibody (goat anti-mouse IgG, AlexFluor488) was added at 1:2000 for 1h. Fluorescence was measured at 485/528 on plate reader, readings were adjusted to negative control (secondary ab only). Wells were washed 3x in PBST between each step.

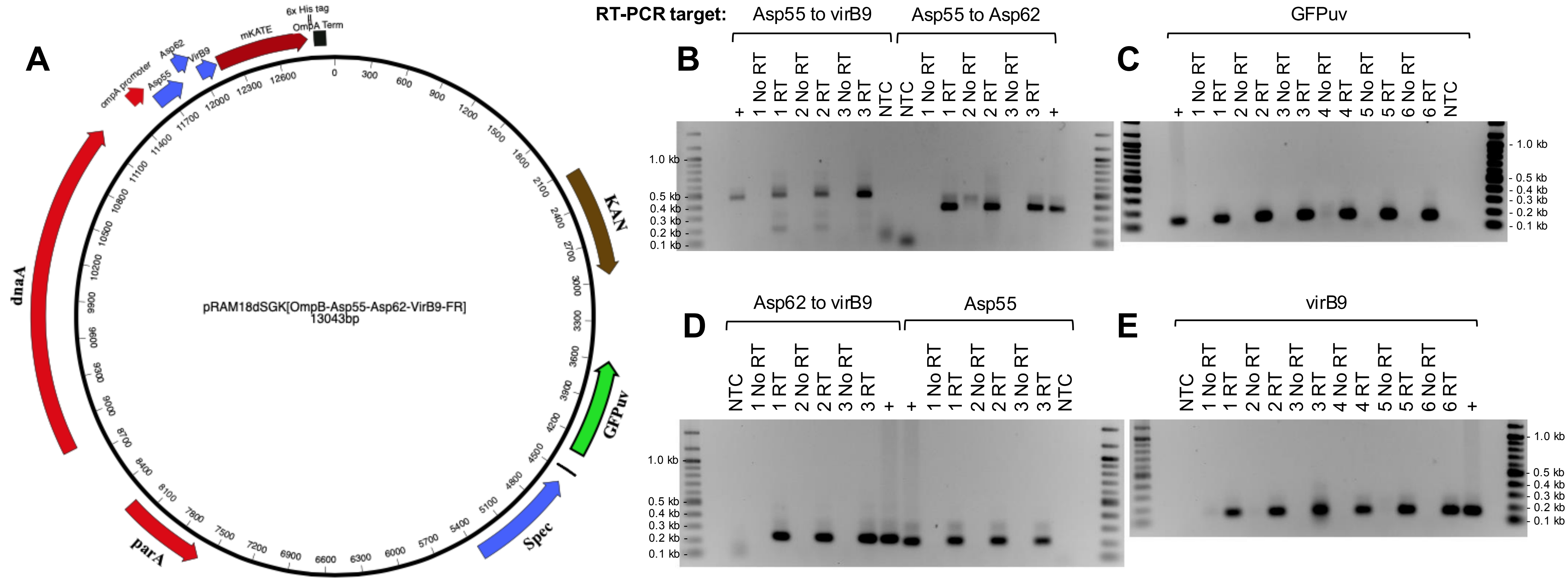

F

+	DNA from *Rp* pRAM18dSGK[OmpA-YchF-Asp55-Asp62-virB9]
NTC	No template control (water)
1	*Rp* pRAM18dSGK [OmpA-Asp55-Asp62-virB9-mKate]
2	*Rp* pRAM18dSGK [OmpA-Asp55-Asp62-virB9]
3	*Rp* pRAM18dSGK [OmpB-Asp55-Asp62-virB9]
4	*Rp* pRAM18dSGK [OmpA-virB9]
5	*Rp* pRAM18dSGK [OmpA-virB9-mKate]
6	*Rp* pRAM18dSGK [OmpB-virB9]

Figure S2: (A) Map of pRAM18dSGK plasmid expressing all epitopes (without YchF). (B-E) Gels of RT-PCR analysis that demonstrate transcription of epitope arrays from a shuttle plasmid transformed into *R. parkeri,* indicating *R. parkeri* is able to express them. The promoter and epitopes are indicated by the sample numbers above the wells and detailed in the table (F). No RT = control lacking reverse transcriptase; NTC = no template control using water instead of RNA; RT = test condition using RNA and reverse transcriptase; + = reactions used DNA as a positive control.

Figure S3: Persistence of vaccine strain *R. parkeri* (G8::lox[virB9-mKate]) in mice. Mouse tissues from vaccinated and boosted mice on day 3 (3 days after primary vaccination), day 35 (7 days after booster), and day 49 (21 days after booster; *Ap*-challenged group shown) were tested by qPCR against rickettsial *gltA* .

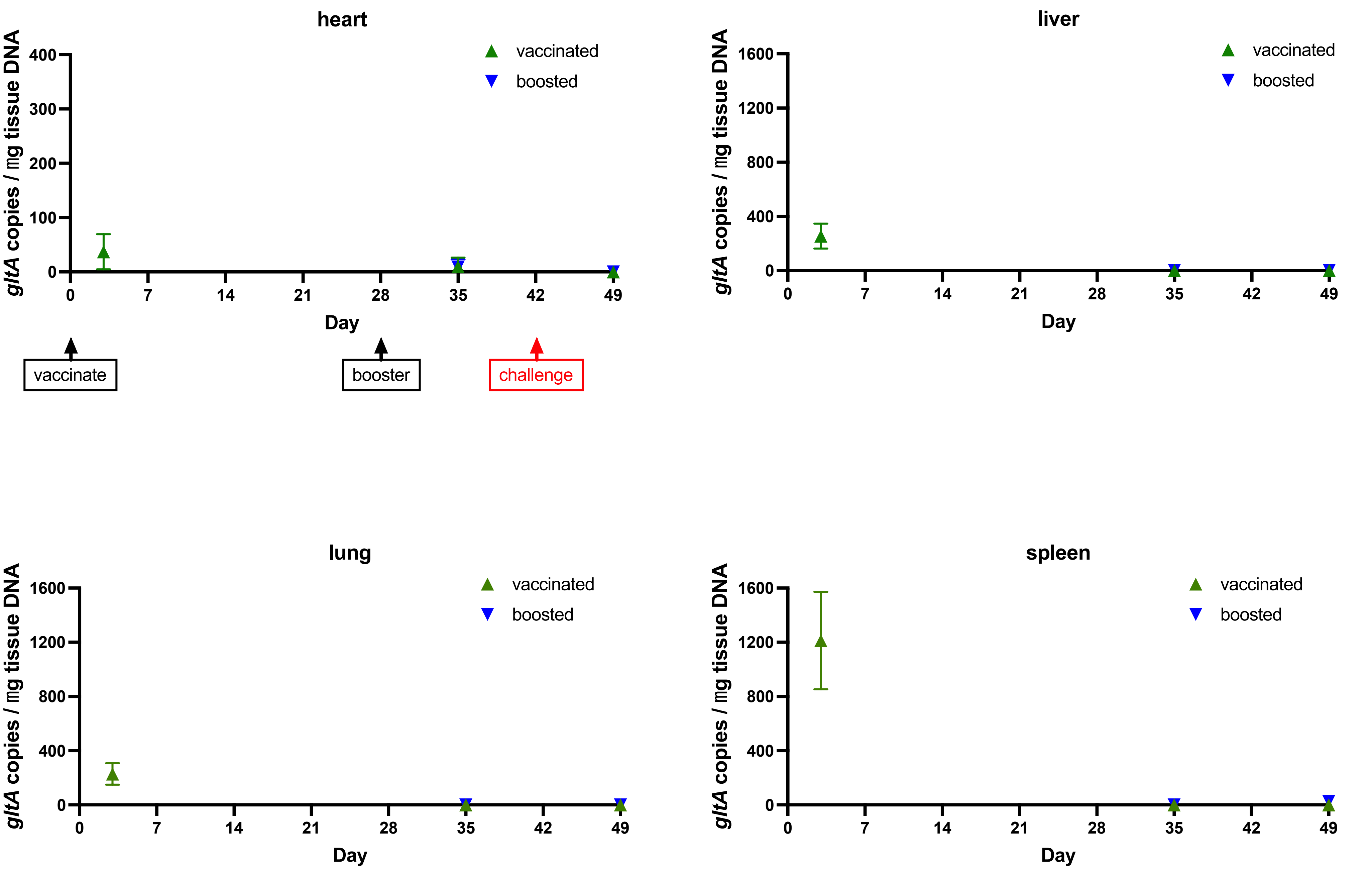

Supplement: Supplementary file 1 [file vaccines-13-00109-s001.zip › Supplementary figures 1-3.pdf]
